# Contrasting evolutionary patterns of spore coat proteins in two *Bacillus* species groups are linked to a difference in cellular structure

**DOI:** 10.1186/1471-2148-13-261

**Published:** 2013-11-27

**Authors:** Hong Qin, Adam Driks

**Affiliations:** 1 Department of Biology, Spelman College, Atlanta, GA 30314, USA; 2 Department of Microbiology and Immunology, Loyola University Medical Center, Maywood, IL 60153, USA

**Keywords:** *Bacillus*, Spore coat, Phylogenetic profiles

## Abstract

**Background:**

The *Bacillus subtilis*-group and the *Bacillus cereus*-group are two well-studied groups of species in the genus *Bacillus*. Bacteria in this genus can produce a highly resistant cell type, the spore, which is encased in a complex protective protein shell called the coat. Spores in the *B. cereus*-group contain an additional outer layer, the exosporium, which encircles the coat. The coat in *B. subtilis* spores possesses inner and outer layers. The aim of this study is to investigate whether differences in the spore structures influenced the divergence of the coat protein genes during the evolution of these two *Bacillus* species groups.

**Results:**

We designed and implemented a computational framework to compare the evolutionary histories of coat proteins. We curated a list of *B. subtilis* coat proteins and identified their orthologs in 11 *Bacillus* species based on phylogenetic congruence. Phylogenetic profiles of these coat proteins show that they can be divided into conserved and labile ones. Coat proteins comprising the *B. subtilis* inner coat are significantly more conserved than those comprising the outer coat. We then performed genome-wide comparisons of the nonsynonymous/synonymous substitution rate ratio, dN/dS, and found contrasting patterns: Coat proteins have significantly higher dN/dS in the *B. subtilis*-group genomes, but not in the *B. cereus*-group genomes. We further corroborated this contrast by examining changes of dN/dS within gene trees, and found that some coat protein gene trees have significantly different dN/dS between the *B subtilis*-clade and the *B. cereus*-clade.

**Conclusions:**

Coat proteins in the *B. subtilis*- and *B. cereus*-group species are under contrasting selective pressures. We speculate that the absence of the exosporium in the *B. subtilis* spore coat effectively lifted a structural constraint that has led to relaxed negative selection pressure on the outer coat.

## Background

The defining feature of bacteria of the family *Bacillaceae* (and the genus *Bacillus* in particular) is the ability to form a specialized alternate cell type, the spore, which can withstand a wide range of environmental stresses, including toxic chemicals, heat, ultraviolet radiation and microbial predation [1-4]. The spore is essentially metabolically dormant and can remain in this state for extreme periods of time. Nonetheless, the spore can return to active growth once nutrient is available, in a process called germination [5]. The ability of spores to remain dormant for long time periods and to resist extreme conditions has made this cell type a major model for studies of cellular defenses against stress.

The *Bacillaceae* thrive in essentially all environments, and have significant taxonomic and phylogenetic diversity, neither of which are fully characterized [6]. The vast majority of research on these organisms has focused on only two *Bacillus* clades. The first of these is the *B. cereus*-group, which is comprised of the closely related species *Bacillus anthracis*, *Bacillus cereus*, *Bacillus thuringiensis*, *Bacillus mycoides*, *Bacillus pseudomycoides* and *Bacillus weihenstephanensis*[7]. Of these, the best studied are *B. anthracis*, the causative agent of anthrax [8], *B. cereus*, an important food-borne pathogen [9], and *B. thuringiensis*, which can produce an insect toxin and, therefore, be used for agricultural biocontrol [10]. The second clade is comprised of *Bacillus subtilis* and its close relatives, including *Bacillus lichenniformis*, *Bacillus pumilus*, *Bacillus amyloliquefaciens*, *Bacillus atrophaeus*, *Bacillus mojavensis*, and *Bacillus vallismortis*. Of these, only *B. subtilis* has received extensive study, making this species the primary model for Gram-positive bacteria and a major model for bacterial development [11]. Because the *B. cereus*-group and *B. subtilis*-group species comprise only a very small subset of the total diversity of the *Bacillaceae*[12], the biology of the majority of these organisms remains poorly understood [13].

A structure found in spores of all *Bacillaceae* (and, indeed, Clostridia as far as is known) is the coat, a protein shell that encapsulates and protects the spore [14-18]. In species where it is the outermost spore structure (see below), the coat has the important role of interacting directly with the environment. For example, proteins on the coat surface play a critical role in the adhesive properties of the spore [19]. It is likely that there are other roles for coat interactions with the environment but they remain undescribed [15,19-23]. The coat has additional diverse functions, including roles in germination and resistance to environmental stresses, like small reactive molecules, degradative enzymes, microbial predation and UV radiation [1,15,20,21,23,24]. It is plausible that any or all of these coat functions could differ among *Bacillaceae* species that inhabit various niches and the challenges faced by these spores may vary as well. These characteristics are among those making bacterial spores unique in nature and have motivated over 140 years of research [11,25,26].

The coat varies significantly in structure among species [15,27-29]. In *B. subtilis*, the coat has three major layers distinguishable by thin-section electron microscopy: a lightly staining inner coat and a darkly staining outer coat that encases a crust [30,31]. The crust is a recently identified structure that is distinct from the outer coat [31]. The composition of the crust is incompletely characterized and it is unknown whether it has functions that are distinct from the other coat layers. Other species, including those of the *B. cereus*-group, have a thinner coat [28]. The coat can also possess more complex features, such as the long filamentous structures in *Bacillus clausii*[29]. *B. cereus*-group species, as well as other species including *B. megaterium*, *B. laterosporus* and *B. vedderi*, possess an additional structure that surrounds the coat, called the exosporium which also varies in structure among species [14,29,32,33]. The exosporium is distinguished from the coat by an apparent gap called the interspace [34]. In *B. cereus-group* species, where it is best studied, the exosporium is comprised of a basal layer from which project a series of fine hair-like projections, referred to as a nap [35]. The composition of the exosporium is not fully known. Several exosporium proteins have been identified, of which the collagen-like glycoprotein BclA is the best characterized [36-38]. The exosporium is known to have roles in interacting with environmental surfaces and other cells [19,39,40]. Importantly, the exosporium is not an impermeable barrier, as it allows passage of small molecules such as sugars and amino acids [41].

Understanding the forces that guide the evolution of the coat can provide unique insight into coat function and formation. For example, identifying highly conserved coat proteins may reveal those with important functions in coat assembly and function [16]. This information, in turn, can help identify which coat proteins are more involved in adaptation. This is an especially interesting question given that the majority of the morphological variation among *Bacillus* spores is in the coat (as well as the exosporium) [27-29]. Importantly, by measuring the degree of selection on a coat protein, it may be possible to show that coat proteins have evolutionarily important roles even when the corresponding coat protein gene mutants lack a detectable phenotype in the laboratory [17,42].

In this work, we aim to test the hypothesis that differences in spore structures can influence the spore coat protein divergence during evolution. We curated a list of *B. subtilis* spore coat proteins, and identified their orthologs based on phylogeny in a group of *Bacillus* species (10 fully-sequenced and 1 partially-sequenced). We then performed a detailed analysis of the molecular evolution of these proteins. Our results showed that evolutionary differences in spore coat proteins can reflect their locations in spore coat layers and differences in spore structure across species.

## Results and discussion

**Figure 1:**
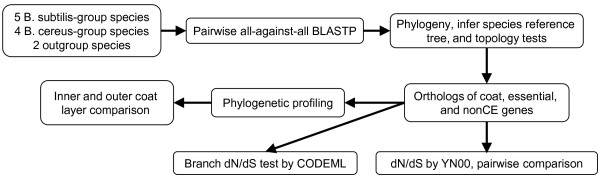
**Overview of the workflow in this study.** Boxes represent the major steps of the computational analysis.

We started with curation of a list of coat proteins and identification of their orthologs in 11 *Bacillus* species by phylogenetic congruency. To investigate whether spore structural diversity influenced coat protein evolution, we then compared conservation of protein compositions in the inner and outer coat layers, compared selection pressures of coat proteins genes with others, and finally studied how selection pressure changes along evolutionary branches within gene trees (Figure 1).

### Identification of orthologs

**Figure 2:**
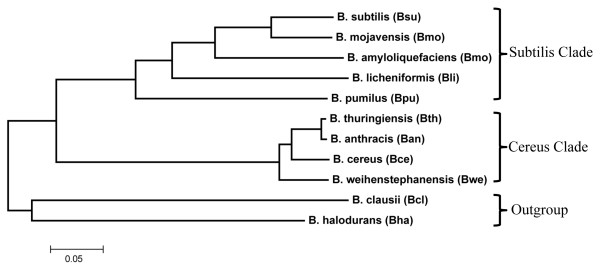
**The species reference tree of the *Bacillus *species under study based on 34 concatenated essential genes.** The Newick format of this tree is (((Bpu,(Bli,(Bam,(Bmo,Bsu)))),(Bwe,(Bce,Ban,Bth))),(Bha,Bcl)). The evolutionary history was inferred using the neighbor-joining method. The optimal tree with the sum of branch length = 1.58 is shown. The percentages of replicate trees in which the associated taxa clustered together in the bootstrap test (1000 replicates) are shown next to the branches. The tree is drawn to scale, with branch lengths in the same units as those of the evolutionary distances used to infer the phylogenetic tree. The evolutionary distances were computed using the JTT matrix-based method and are in the units of the number of amino acid substitutions per site. All positions containing gaps and missing data were eliminated from the dataset. Phylogenetic analyses were conducted in MEGA4.

A defined species reference tree is important in phylogenetic analysis [43,44]. However, species trees of bacteria are difficult to construct [45]. The *B. cereus* sensu lato group is known to be very closely related. Sequence variations suggest that the *B. cereus* sensu lato group is a group of asexual clonal lineages [46]. *B. cereus* is also known to be an intermingled cluster of genetically diverse strains [47]. To facilitate appropriate molecular evolution analysis, we chose in this study to infer a species reference tree using only the fully sequenced genomes of species type strains. We used the concatenated sequences of 34 essential genes and generated a species reference (Figure 2), which is consistent with the 16S rRNA gene tree and previous reports (see Methods). Given that many bacterial gene trees may differ from the species reference tree, we tested alternative tree topologies and found that alternative branching patterns within the two major clades are mostly acceptable (see Methods).

Previous work shows there are likely more coat proteins in *B. subtilis* than the 50 or so that have been relatively well characterized [18,48]. Using sequence similarity criteria, and data from microarrays studies identifying genes of unknown function that are expressed late in sporulation [49,50], we compiled an expanded list of 73 genes (see Additional files 1 and 2), that includes genes we regard as strong candidates for coat protein genes [48,49]. Previous studies strongly suggest that these criteria have a high likelihood of identifying novel coat protein genes [18,48]. Over 80% (60 out of 73) of these genes were annotated as spore coat protein genes independently by another group [14].

We performed pairwise all-against-all BLASTP searches [51] for all studied genomes (Additional file 1: Table S1). Potential orthologs were identified both by Markov clustering (MCL) [52] and reciprocal best hits (RBH) [53,54]. We iterated the Inflation parameter (I) of MCL from 1.1 to 8.0 to explore the granular effect on gene clusters. For the 73 coat protein genes, we found that I = 3.1 is the smallest value that can give the largest number of orthologous groups in the coat protein genes, which was 70 clusters (3 clusters contain duplicates). We distinguished orthologs from paralogs by comparing the bootstrapped neighbor-joining trees of the candidate orthologs to the species reference trees and its alternatives. Examination of the multiple sequence alignments showed that many unresolved gene trees were due to repeat sequences, also known as low complexity regions (LCRs), in coat proteins. Because some coat proteins tend to contain a substantial number of LCRs, filtering them out during BLASTP searches would result in a reduction of detectable hits [55]. To avoid this problem, we included LCRs during BLASTP searches, used bootstrapping to average out the peculiar topologies due to repeat-caused alignment problems, compared the topology between gene trees and the species trees, and excluded the topological inconsistent hits as ‘false positives’. For gene families with gene loss, a pruned reference tree was used. In addition, all of the phylogenies of coat protein orthologous groups were double-checked visually. This visual examination led to the identification of a split ORF in one coat protein gene (see the section of improved annotation in the Additional file 1).

Among the 73 coat proteins in *B. subtilis*, six were closely related paralogs that could not be separated into orthologous groups. Hence, we obtained 70 orthologous clusters (three clusters contain two orthologous groups). The pair BG13471 (CotU) and BG10492 (CotC) are so similar that their orthologs in *B. licheniformis* were arbitrarily chosen for further analysis.

For orthologous identification of non-coat protein genes, only automated analyses were used, but LCRs were filtered out during BLASTP searches to improve specificity.

### Phylogenetic profiling of spore coat proteins

Analysis of the distributions of protein orthologs among species, i.e. the phylogenetic profile, can give important insights into protein evolution and help identify those proteins with essential functional roles. Previous profile analyses of coat protein genes were based on sequence similarity approaches [14]. Because orthologs are genes in different species that are derived from a single ancestral gene [56], an orthologous relationship is by definition determined by phylogeny, using molecular evolutionary measures of gene distances [57].

**Figure 3:**
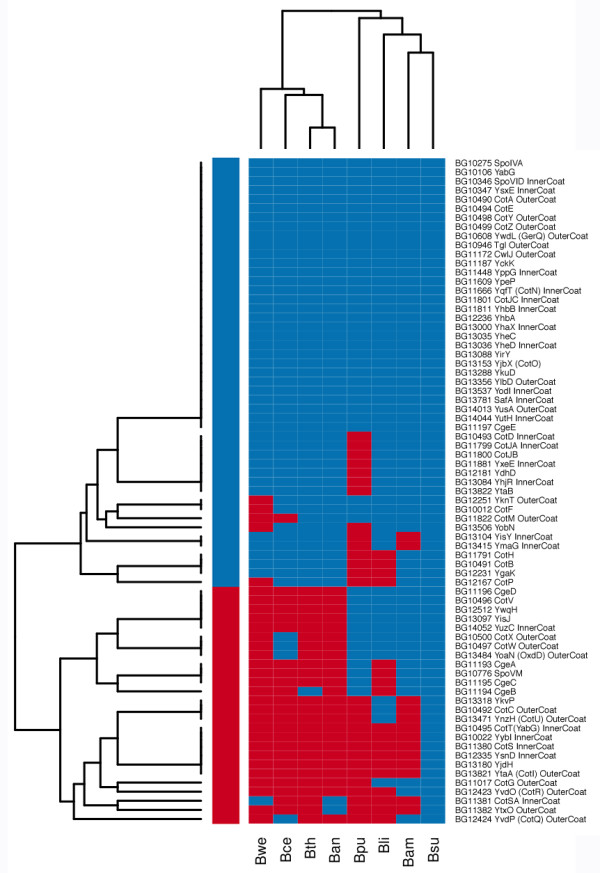
**Distribution of orthologous hits of coat proteins in the two major *Bacillus *clades.** Blue indicates ‘1’ (the presence of orthologous hits) and red indicates ‘0’ (the absence of detectable orthologous hits). Hierarchical clustering using average linkage and hamming distances was applied both by rows, which led to grouping of coat proteins into ‘conserved’ and ‘labile’ categories. Clustering by columns led to grouping of species that agrees with the species reference tree.

We used a phylogeny-based approach to identify orthologous distributions of coat proteins (the set of coat protein orthologs among species) in 11 *Bacillus* species. The resulting coat protein phylogenetic profiles suggest that coat protein genes can be partitioned into evolutionarily conserved and labile ones (Figure 3). The orthologous distribution for each coat protein orthologous group (named after the *B. subtilis* (Bsu) gene IDs) was generated by assigning 1 to each species with detectable orthologous hits and assigning 0 otherwise. The dissimilarities in the coat protein orthologous distributions are strong enough that their clustering result by species agrees with the species reference tree in Figure 2. For comparison, essential genes of *B. subtilis* are mostly conserved in the studied genomes (Additional file 1: Figure S1).

### Protein composition of the *B. subtilis* inner coat is more conserved than the outer coat

We speculated that proteins comprising the outermost structures of the spore would be more evolutionarily labile, since these proteins would be most likely to make direct contact with the environment. If so, this lability might be reflected in the coat protein gene phylogenetic profiles. Specifically, we expected to find that coat proteins closer to the spore surface would be more labile than coat proteins at more interior locations. To test this hypothesis, we first analyzed the phylogenetic profiles of the coat proteins in *B. subtilis*, because it is already known that many if not most of the outermost proteins in *B. subtilis* are among the already identified outer coat proteins (or outer coat protein candidates) [14,17,48,58]. We note that proteins designated in the literature or in genome annotations as members of the outer coat could also be present in the crust, the recently identified and still poorly characterized coat layer surrounding the outer coat [31]. Although, in the present study, we chose to avoid confusion with the existing literature by retaining the designation “outer coat proteins” to refer to any coat proteins in layer(s) surrounding the inner coat, we emphasize that future studies are likely to assign at least some of them to the crust, in addition to or instead of the outer coat.

We first tested whether the coat protein phylogenetic profiles were associated with their known (or likely) sub-locations within inner or outer coat layers by constructing a two-by-two table and then analyzing the statistical associations (Table 1). The conserved coat proteins in the *B. cereus*-group are those with orthologous hits in all four species, and the labile coat proteins in the *B. cereus*-group are those missing at least one orthologous hit in the *B. cereus*-group. Consistent with our hypothesis, 17 out of 23 inner coat proteins are conserved in the *B. cereus*-group, while only 8 out of 20 outer coat proteins are conserved in this group (one sided Fisher-exact test, p = 0.026).

**Table 1:** Protein composition of inner coat is more conserved than that of outer coat

**Coat proteins**	**Inner coat**	**Outer coat**
Conserved in the *B. cereus*-group	17	8
Labile in the *B. cereus*-group	6	12
Subtotal	23	20
One sided Fisher’s exact test, p-value = 0.026		

A two-by-two table is generated based on the spore coat location for proteins and their orthologous profile. The “conserved” coat protein genes are those with orthologous in all of the four species in the *B. cereus*-group, as shown in Figure 2. The “labile” coat protein genes are those with at least one missing orthologous hit in the *B. cereus*-group.

**Figure 4:**
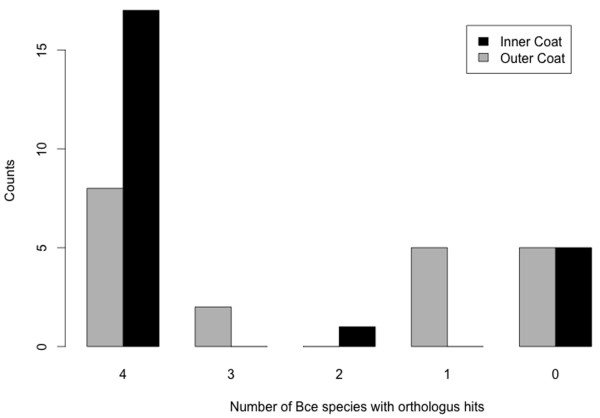
**Histograms of orthologous hits for *B. subtilis *inner and outer coat proteins.** The number of orthologous hits for each coat protein in the *B. cereus*-group is calculated as the sum of the hits in *B. anthracis*, *B. thuringiensis*, *B. cereus*, and *B. weihenstephanensis*. Each bin represents the number of *B. subtilis* coat proteins with the indicated number of orthologous hits.

We are aware that the test in Table 1 can be influenced by the partitioning of coat proteins into conserved and labile categories. To avoid this caveat, we examined the orthologous hits directly. For each coat protein, we counted the number of *B. cereus* group species that contains an orthologous hit based on their phylogenetic profile in Figure 3. Histograms of these counts are plotted side-by-side for inner and out proteins in Figure 4. The inner coat proteins have significantly more orthologous hits than the outer coat proteins (Wilcoxon test, p = 0.039).

Based on the above two analyses, we concluded that protein compositions are more conserved in the inner coat than the outer coat between the *B. subtilis*-group and *B. cereus*-group species. We speculate that in all the species analyzed above, the relatively greater lability of the outer layer protein composition is due to an important role for this layer in adaptation to specific niches. It is possible that the adaptive features of the outer coat layer is a consequence of many coat protein working together, for example, by contributing a particular chemical property to the spore surface [19]. These adaptive changes of cellular structures can include positive selection, relaxed negative selection, and loss of negative selection at gene levels. The loss of negative selection on some genes is consistent of their absence of orthologous hits in some species.

### Relatively higher dN/dS ratios of coat protein genes in the *B. subtilis* group

If the diversity in *Bacillaceae* spore coat morphology reflects adaptation of these species to a range of environments, then we may be able to detect signatures of selection from the perspective of molecular evolution. We chose to address this by estimating the ratio of non-synonymous (dN) to synonymous (dS) substitution rates, ω, a proxy for selective pressure [59]. An increase in ω can suggest a relatively faster non-synonymous substitution rate, after adjusting for mutational background, due to either relaxed negative selection or positive selection in divergent species [59].

**Figure 5:**
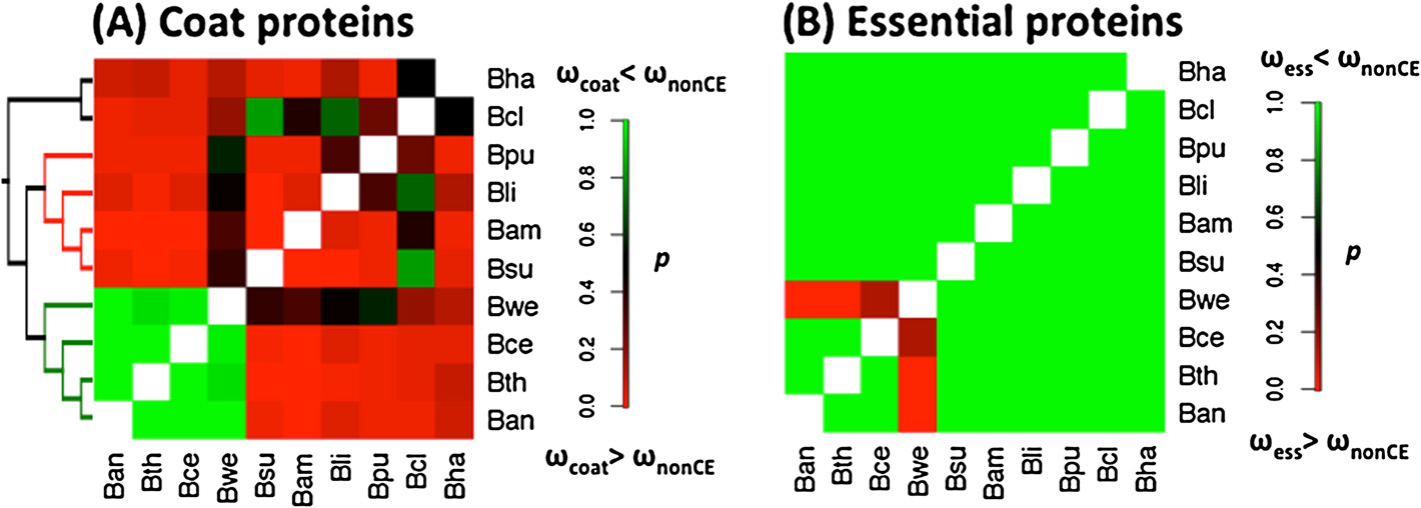
**Heat map presentations for all pairwise Wilcoxon tests in the ten fully sequenced species. (A)** Pairwise tests between coat proteins genes and non-coat non-essential genes. Each cell represents a one-sided p-value calculated with the alternative hypothesis: coat protein gene ω > nonCE ω. Most cells within the *B. subtilis*-group are red with p-values less than 0.05, indicating coat protein gene ω > nonCE ω. Most cells within the *B. cereus*-group are green with p-values greater than 0.95, indicating the opposite: coat protein gene ω < nonCE gene ω. The diagonal cells would suggest self-comparison and are excluded. A species reference tree was drawn on the left. **(B)** Pairwise tests between essential genes and nonCE genes. Each cell represents a one-sided p-value calculated with the alternative hypothesis: essential gene ω > nonCE ω. Essential genes generally have smaller ω and evolve slower than nonCE genes. An exception occurs in the *B. weihenstephanensis* branch, where essential genes have higher ω than nonCE genes. In the panels, *p* represents p-values*,* ω_coat_, ω_nonCE_, and ω_ess_ represent ω for coat, nonCE, and essential proteins genes. Explanations of abbreviated species names are in Figure 2.

First, we tested whether coat protein genes tend to have higher or lower ω in comparison to other protein genes. For comparison, we chose the reference gene group as the remaining genes in a genome after excluding coat and essential genes, referred to as non-coat non-essential (nonCE) genes. We used YN00 [60] to estimate ω for all genes based on pairwise alignments of ortholog pairs in the 10 species with complete genomes. Two-sample Wilcoxon tests were performed between the list of coat protein genes and the list of nonCE genes in all possible pairwise combinations of the 10 species (Figure 5A). We calculated p-values using the one-sided test with the alternative hypothesis: coat ω > nonCE ω. Hence, small p-values (red color) indicate coat protein genes tend to have higher ω than nonCE genes (Figure 5A). Although simple pairwise comparisons usually cannot narrow down evolutionary events to specific branches, the matrix approach used here can detect differences between clades. In Figure 5A, the patterns in the *B. subtilis*-group and the *B. cereus*-group are clearly opposite. In the *B. subtilis*-group, the p-values are mostly less than 0.05, and coat protein genes show higher ω than do nonCE genes. In the *B. cereus*-group, the p-values are mostly greater than 0.95, which means coat ω < nonCE ω is observed. Hence, the patterns of coat protein gene evolution differ between the *B. subtilis*- and *B. cereus*-groups. These contrasting ω patterns held when additional *B. cereus* genomes were included in the analysis (Additional file 1: Figure S2A). As expected, the contrasting evolutionary patterns of coat protein genes are not pronounced in pairwise tests of dN measures (Additional file 1: Figure S2B), and are absent in pairwise tests of dS measures (Additional file 1: Figure S2C). For comparison, the ω of essential genes are significantly lower than those of nonCE genes (with an exception in the *B. weihenstephanensis* lineage) (Figure 5B and Additional file 1: Figure S2D), further validating this pairwise matrix approach. These results show that negative selection pressure on coat protein genes is significantly stronger in the *B. cereus*-group than in the *B. subtilis*-group.

**Figure 6:**
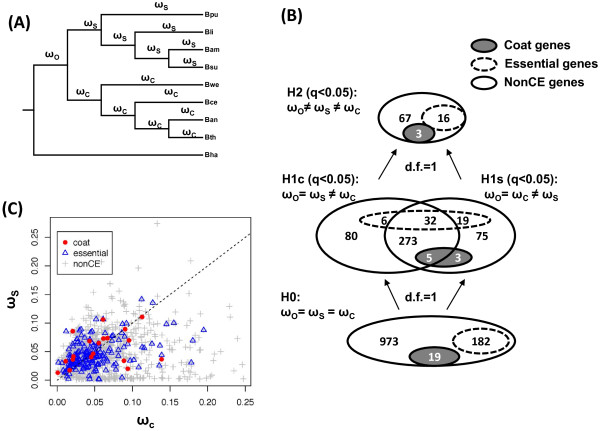
**Nested model test on differential selective pressures between the *B. subtilis*-group and the *B. cereus*-group in conserved genes. (A)** Specification of ω along branches. **(B)** Summary of nested model test results in Venn diagrams. The numbers of coat proteins are indicated by closed circles in gray, and the numbers of essential genes by dashed circles in white. There are a total of 1174 genes (including 19 coat protein and 182 essential genes) in this LRT study. Alternative models of H1c, H1s, and H2 were accepted with q-values less than 0.05. **(C)** Comparison of branch ω in the *B. subtilis* clade (ω_s_) and the *B. cereus* clade (ω_c_). The dash diagonal line indicates ω_s_ = ω_c_. Red circles represent coat protein genes, open triangles essential genes, and gray crosses are nonCE genes. Estimations of branch ω were based on model H2. For coat proteins, ω_s_ > ω_c_ was found with a p-value of 0.072 based on paired Wilcoxon test.

Second, we investigated how ω varies between the two major clades within each gene tree. Comparison within gene trees offers an alternative approach to the pairwise comparisons across genes. We calculated the likelihood of different evolutionary scenarios, designed in nested branch models in CODEML, and applied likelihood ratio tests (LRTs) [43]. We are aware that the nested model test approach detects changes only within each gene tree (not between two different groups of genes), and is, therefore, more conservative than the pairwise analysis. Meaningful LRTs should be calculated using the same mathematical model, i.e, the same tree topology, which constrained us to focus LRTs on conserved genes. We selected 1174 conserved gene families whose neighbor-joining gene trees agree with the species reference tee, and also contain an orthologous hit in the outgroup *B. halodurans*. These conserved gene families include 19 coat protein genes and 182 essential genes. We then calculated their likelihood for four nested branch models: H0, H1c, H1s, and H2 using CODEML (Figure 6A) [43]. The results, at a false-discovery rate of 0.05 (q-value = 0.05), are summarized in Venn diagrams (Figure 6B). We found that 396 genes (including 5 coat protein genes) show significantly different ω values in the *B. cereus*-group (model H1c), and 407 genes (including 8 coat protein genes) show significantly different ω values in the *B. subtilis*-group (model H1s). The results here also suggest that differential evolution of coat proteins between the *B. subtilis*-group and *B. cereus*-group occurred in concert with many other genes. In other words, changes in the coat are likely part of large-scale changes between the two species groups. We then compared the branch ω in the *B. subtilis*-group, ω_s_, and the *B. cereus* group, ω_c_ (Figure 6C). For the 19 coat protein genes, the alternative hypothesis ω_s≥_ω_c_ was found with a p-value of 0.072, which is in general agreement with the pairwise analysis in Figure 5.

In the interpretations just described, we have assumed that ω is an accurate reflection of the strength of selection. However, other interpretations are possible. The genomes of the *B. cereus*-group are relatively closely related, whereas genomes in *B. subtilis*– group species are more divergent. In closely related bacteria, increased ω are often observed, which can be attributed to changes in effective population size, relaxation of negative selection, differences in divergence time, or limitations of parametric evolution models [61]. For closely related genomes of asexual organisms, negative selection will not have enough time to “purify” the deleterious mutations and thereby leads to relatively high ω. This is similar to the mistreatment of standing polymorphism as fixed changes in diploid sexual organisms. This problem is at least partially due to a bias in current genome sequencing efforts towards those genomes with perceived medical relevance. Moreover, it is important to emphasize that species identification remains a commonly encountered and significant challenge in bacterial genome analysis. Species misidentification can lead to mistreating polymorphism as divergence which, in turn, leads to false-positive signatures of selection. We have sought to mitigate this problem by focusing on the genomes of well-established species-type strains. We are aware that genomes of many more *Bacillus* strains have been sequenced recently. However, most of these are assigned to the species that have been studied here, and nucleotide changes in many of these genomes should be treated as polymorphisms.

The low ω values in most coat proteins indicate that most residues in their sequences are under purifying selection [62]. Consequently, even though only a small fraction of coat protein gene mutations have phenotypes that are readily detectable in the laboratory [15,29], most or all coat proteins likely contribute to the overall fitness of the spore. We were unable to find a correlation between the known phenotype of each coat protein gene mutation and its degree of conservation. However, this is not surprising, as coat protein gene mutants are rarely if ever analyzed using ecologically realistic assays [17]. Interestingly, many coat proteins have a significant proportion of disordered regions (see supporting information). Protein structures are known to correlate with the coding sequence evolution [62]. It is plausible that disordered regions of coat proteins may contribute to the contrasting sequence substitution patterns between the two *Bacillus* groups, through their roles in spore coat assembly.

## Conclusions

**Figure 7:**
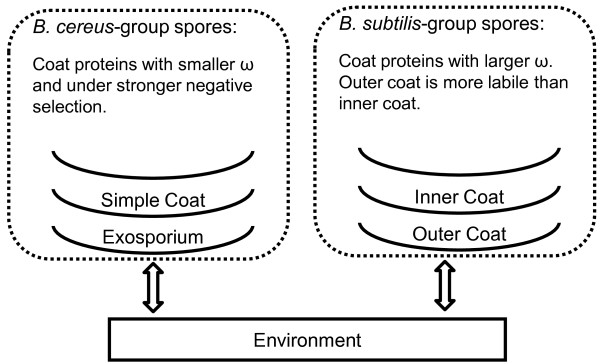
**Cell structural differences in outer-spore layers are plausible causes for the observed evolutionary differences in coat protein genes between the *B. subtilis*-group and *B. cereus*-group.**

We demonstrated a strong association between the structural diversity of the coat and the evolutionary patterns of its protein components between the *B. subtilis*-group and *B. cereus*-group (Figure 7), by two lines of evidences: First, in *B. subtilis*, protein composition of the inner coat is more conserved than that of the outer coat based on phylogenetic profiles (Table 1 and Figure 4); Second, coat protein genes have significantly higher ratio of nonsynonymous versus synonymous substitution rates, dN/dS, than nonCE genes in *B. subtilis*-group but not in the *B. cereus*-group (Figure 5), which is consistent with dN/dS changes within gene trees (Figure 6). Because species in the *B. subtilis*-group lack an exosporium, negative selection on coat protein genes might be relaxed due to the removal of a structural constraint. This is an appealing possibility given the likely importance of the outer coat in the interaction with environment species without exosporia (Figure 7). Even in exosporium-bearing species, the coat still makes significant (albeit indirect) contact with the environment, since the exosporium permits diffusion of small molecules. Nonetheless, in the absence of the exosporium, the coat surface likely has direct roles in adhesion to surfaces in the environment. As already discussed, *B. subtilis* possesses a recently discovered outermost coat layer called the crust, which is composed, at least in part, of proteins presently designated as outer coat proteins [31]. The current ambiguity in assignment of coat proteins to the crust or outer coat layer does not affect the conclusions of our work. However, as the composition of the crust becomes clarified in future studies, we may learn that its evolutionary history has features that distinguish it from the true outer coat.

Our work raises several intriguing questions for future studies. First, what are the broader biological and functional implications of the different evolutionary patterns of coat protein genes among different *Bacillaceae* clades? Second, do exosporium protein genes follow an evolution trend similar to the outer coat in *B. subtilis*, as we would predict? In future studies, we will apply the approach described here to those genes, to determine not only whether they evolve more rapidly than coat protein genes, but also whether different rates of evolution can be detected within the exosporium sublayers.

One of the most interesting consequences of this work is the likely role for the outer coat and crust proteins in variation among spores of the *Bacillaceae*. The phylogenomic approach employed in this study is likely to be very useful to further investigations into the divergent ecological histories and patterns of adaptation among spore-forming bacteria. We hope that this work prompts deeper investigations into poorly studied species with intriguing lifestyles and poorly studied ecological niches [13].

## Methods

### Sequences

Genomes analyzed in this study are summarized in Additional file 1: Table S1. Most of the genomes are the species type-strains. We analyzed 5 *B. subtilis*-group genomes: *Bacillus subtilis subsp. subtilis str. 168*, *Bacillus mojavensis* RO-H-1, *Bacillus licheniformis* ATCC 14580, *Bacillus amyloliquefaciens* FZB42, and *Bacillus pumilus* SAFR-032. We analyzed 6 *B. cereus*-group genomes: *Bacillus anthracis str. Ames*, *Bacillus cereus* ATCC 10987, *Bacillus cereus* ATCC 14579, *Bacillus cereus* E33L, *Bacillus thuringiensis serovar konkukian*, and *Bacillus weihenstephanensis* KBAB4. We used genomes of *Bacillus clausii* KSM-K16 and *Bacillus halodurans* C-125 as outgroups. Genes of the draft genome of *Bacillus mojavensis* RO-H-1 were predicted by GLIMMER [63].

The rRNA sequences were obtained from the Ribosomal Database Project II release 9.56 [64]. The annotation of the *B. subtilis* genome was based on SubtiList [65,66]. Essential genes were parsed out from Kobayashi et al. 2003 [67]. Coat protein genes in *B. subtilis* were annotated in the Driks group. After excluding the coat protein genes and essential genes, the remaining genes are referred to as non-coat non-essential (nonCE) genes. The lists of *B. subtilis* coat protein genes and their locations within the coat layers, if known, are provided in Additional files 1 and 2. The lists of coat essential and nonCE genes in all the studied species are also provided at our GitHub repository.

### Inference of species reference tree and alternative topologies

To infer the species reference tree, we used both the 16S rRNA approach and the multi-locus approach [45]. The 16S rRNA approach has often been used for identification of *Bacillus* species [12,68-70]. Using the Ribosomal Database Project [64], we curated 148 16S ribosomal RNA sequences from *Bacillaceae* and their related species and generated structure-based alignments [71]. *Alicyclobacillus acidocaldariu* and *Geobacillus kaustophilus* were used as outgroups. Phylogenetic trees were generated using neighbor-joining, maximal parsimony and Bayesian approaches [72-76]. Neighbor-joining trees were evaluated by bootstrap [57]. Although the 16S rRNA gene tree is generally in agreement with previous results using the 16S rRNAs [12,69,70], the resulting tree is only partially resolved (Additional file 1: Figure S3).

For the multi-locus approach, we chose a sequence concatenation-based approach [77]. We curated a list of 34 essential genes in *B. subtilis* that had unequivocally single-orthologs in other genomes. We concatenated the coding sequences of these 34 genes into a super-gene of about 36.6 Kb in length for each species-type strain. The neighbor-joining tree of these concatenated sequences is 100% supported by bootstrap resampling and is used as the resolved species reference tree (Figure 2). In this resolved tree, the ATCC 14579 type strain of *B. cereus* is positioned next to *B. weihenstephanensis* KBAB4, and *B. anthracis* and *B. thuringiensis* konkukian are next to each other, which is similar to the neighbor-joining tree based on concatenated sequences of 7 house-keeping genes [78]. This species tree is further supported by our clustering results of the coat protein phylogenetic profiles (Figure 3) and by the CONSEL topology tests in essential genes (Additional file 1: Table S2). *B. thuringiensis konkukian* is also reported to be close to *B. anthracis*[79].

Given that many bacterial genes in a genome can have different gene trees, using only one reference gene tree for ortholog identification can lead to many false negatives. Based on the neighbor-joining trees of individual coat protein genes, we found 9 major topologies in the coat protein genes, excluding the influences of gene duplication, gene-loss, and unresolved trees. Alternative branching patterns frequently occur within the *B. subtilis* and *B. cereus* groups, but not between these two groups. To find out which alternative topologies were statistically acceptable, we estimated their likelihood using CODEML and evaluated them by CONSEL [80] in the 34 essential genes (Additional file 1: Table S2). A total of 10 topologies (including a negative control) were tested using the AU-test provided by CONSEL. Overall, most alternative branching patterns within the two major groups are accepted, but those occurring between the two major clades (such as the 10^th^ tree topology) are consistently rejected at a p-value of 0.05. The species reference tree in Figure 2 (the 1st tree in Additional file 1: Table S2) is ranked as the highest 20 out of 34 times, and is only rejected 1 out of 34 times at a p-value of 0.05. Therefore, for ortholog identification, we accepted trees with alternative branching patterns within the two major clades.

### General computing methods

Statistical analyses and data visualization were largely performed in the R language and environment [81]. Sequence alignments were done by CLUSTALW coupled with BioPerL [82,83]. Neighbor-joining phylogenies were initially inferred for all genes, evaluated by bootstraps in PHYLIP [84] and APE [85]. Topological differences were first identified by TREEDIST from the PHYLIP software package [85]. Likelihoods of different gene trees were estimated by CODEML [43,44,86] and compared by CONSEL [80] (Figure 1). Synonymous and nonsynonymous substitution rates were calculated using YN00 [60] for pairwise comparisons (Figure 1). For nest model tests in CODEML, we used the template control files provided by the lysozyme example [43,87], in which ω values are specified for branches (Figure 1). Drawings of phylogeny were either manually performed in MEGA and Dendroscope [72,73,88] or automated using APE in R. Initial clustering of sequence was done using MCL [52] and PERL scripts. Protein statistics were calculated by PEPSTATS from EMBOSS [89]. Disordered regions in proteins were predicted using DisEMBL [90]. Low complexity regions (LCRs) were calculated using XNU [91]. Handling of sequences and automation were done largely by PERL scripts in conjunction with BioPerl and shell scripts in LINUX/UNIX platforms. A small fraction of Python/BioPython codes were also used, especially for the topological analysis.

## Availability of supporting data

In addition to the supplementary information, we created a GitHub repository, [92]. This GitHub repository contains the full genomes analyzed, the list of annotated coat protein genes, their sequences and alignments, gene trees, running results, and the key PERL and R scripts for data analysis and generations of figures.

## Abbreviations

LRTs: Likelihood ratio tests; LCRs: Low complexity regions; NonCE: Non-coat and non-essential; Bam: *B. amyloliquefaciens*; Ban: *B. anthracis*; Bce: *B. cereus*; Bcl: *B.* clausii; Bha: *B. halodurans*; Bli: *B. licheniformis*; Bmo: *B. mojavensis*; Bpu: *B. pumilus*; Bsu: *B. subtilis*; Bth: *B. thuringiensis*; Bwe: *B. weihenstephanensis.*

## Competing interests

The authors declare that they have no competing interests.

## Authors’ contributions

HQ and AD designed the study, HQ performed the study, and HQ and AD wrote the manuscript. Both authors read and approved the final manuscript.

## Supplementary Material

Additional file 1 Contains Table S1 and S2, Figures S1-S4, improved annotations of spore coat proteins, and list of 34 essential genes. Click here for file

Additional file 2 The list of spore coat protein genes and their orthologs. Click here for file
