# Supplementary material for: Contrasting evolutionary patterns of spore coat proteins in two Bacillus species groups are linked to a difference in cellular structure
Source: BMC Evol Biol. 2013 Nov 27;13:261. doi: 10.1186/1471-2148-13-261 (PMC4219348; doi:10.1186/1471-2148-13-261)
Supplement: Additional file 1 — Contains Table S1 and S2, Figures S1-S4, improved annotations of spore coat proteins, and list of 34 essential genes. [file 1471-2148-13-261-S1.pdf]

Supplementary Information for
Contrasting evolutionary patterns of spore coat proteins in two groups of *Bacillus* species are linked to a cellular structural difference.

Hong Qin and Adam Driks

Contents

Table S1. Key genomes analyzed in this study. 2

Table S2. Topologies tested by CONSEL in 34 essential genes. 3

Figure S1. Phylogenetic profile of essential genes. 4

Figure S2. Heatmap presentations for all pairwise Wilcoxon tests for ω, dN, and dS with additional genomes. 5

Figure S3. One of the maximal likelihood trees of 16S rRNA. 6

Figure S4. Bootstrap consensus tree of the CotS-CotI-YutH family. 7

Improved annotations of *Bacillus* spore coat proteins. 8

List of the 34 essential *B. subtilis* genes for the species reference tree and topology tests 9

References 10

Table S1. Key genomes analyzed in this study.

Genomes	Abbreviations	Clades	Sources
Bacillus amyloliquefaciens FZB42	Bam	Subtilis	Genbank NC_009725
Bacillus anthracis Ames	Ban	Cereus	JCVI CMR
Bacillus cereus ATCC 14579	Bce	Cereus	JCVI CMR
Bacillus cereus ATCC 10987	Bce87	Cereus	JCVI CMR
Bacillus cereus ATCC E33L	Bce3L	Cereus	JCVI CMR
Bacillus clausii KSM-K16	Bcl	Outgroup	JCVI CMR
Bacillus halodurans C-125	Bha	Outgroup	JCVI CMR
Bacillus licheniformis ATCC14580	Bli	Subtilis	JCVI CMR
Bacillus mojavensis RO-H-1	Bmo	Subtilis	JCVI CMR
Bacillus pumilus SAFR-032	Bpu	Subtilis	Genbank NC_009848
Bacillus subtilis 168	Bsu	Subtilis	SubtiList
Bacillus thuringiensis konkukian	Bth	Cereus	JCVI CMR
Bacillus weihenstephanensis KBAB4	Bwe	Cereus	Genbank NC_010184

Clades indicate the phylogenetic grouping for each genome. SubtiList: http://genolist.pasteur.fr/SubtiList/ [1]; JCVI CMR: http://cmr.jcvi.org/. Our analysis of the partial genome of *Bacillus mojavensis* RO-H-1 is limited to coat protein orthologs. Except Bce87 and Bce3L, all other genomes are type strains for their represented species.

Table S2. Topologies tested by CONSEL in 34 essential genes.

No.	Trees in Newick format	AU test summary by CONSEL	
1	((Bha,Bcl), (Bpu,(Bli,(Bam,(Bmo,Bsu)))), (Bwe,(Bce,(Ban,Bth))));	Ranked highest in 20 genes.	Accepted in 33 genes
2	((Bha,Bcl), (Bpu,(Bli,(Bam,(Bmo,Bsu)))), ((Bwe,Bce),(Ban,Bth)));	Ranked highest in 7 genes.	Accepted in 33 genes
3	((Bha,Bcl), (Bpu,(Bli,(Bam,(Bmo,Bsu)))), (Bwe,(Bth,(Bce,Ban))));		Accepted in 5 genes
4	((Bha,Bcl), ((Bpu,Bli),(Bam,(Bmo,Bsu))), (Bwe,(Bth,(Bce,Ban))));		Accepted in 7 genes
5	((Bha,Bcl), ((Bpu,Bli),(Bam,(Bmo,Bsu))), ((Bwe,Bth),(Bce,Ban)));		Accepted in 4 genes
6	((Bha,Bcl), (Bli,(Bpu,(Bam,(Bmo,Bsu)))), (Bwe,(Bce,(Ban,Bth))));	Ranked highest in 6 genes.	Accepted in 26 genes
7	((Bha,Bcl), (Bli,(Bpu,(Bam,(Bmo,Bsu)))), ((Bwe,Bce),(Ban,Bth)));	Ranked highest in 1 gene.	Accepted in 22 genes
8	((Bha,Bcl), (Bam,(Bli,(Bpu,(Bmo,Bsu)))), (Bwe,(Bce,(Ban,Bth))));		Accepted in 5 genes
9	((Bha,Bcl), (Bam,(Bpu,(Bli,(Bmo,Bsu)))), (Bwe,(Bce,(Ban,Bth))));		Accepted in 4 genes
10	((Bha,Bcl), (Bwe,(Bli,(Bam,(Bmo,Bsu)))), (Bpu,(Bce,(Ban,Bth))));	Ranked lowest in 33 genes.	Accepted in 0 genes

The first tree is the neighbor-joining tree based on the concatenated sequences. The first tree is the more representative one and is chosen as the species reference tree. Key differences between the first tree and other topologies are underlined. Alternative branching patterns with the *B subtilis*-clade or *B cereus*-clade were often accepted, and these are treated as acceptable alternative topologies during identification of orthologs. Alternative branching patterns violating the two major clades, such as in the 10th tree, was consistently rejected. Data and codes used to perform the tests are at `https://github.com/hongqin/BacillusSporeCoat/tree/master/tree.test/topology.tests`.

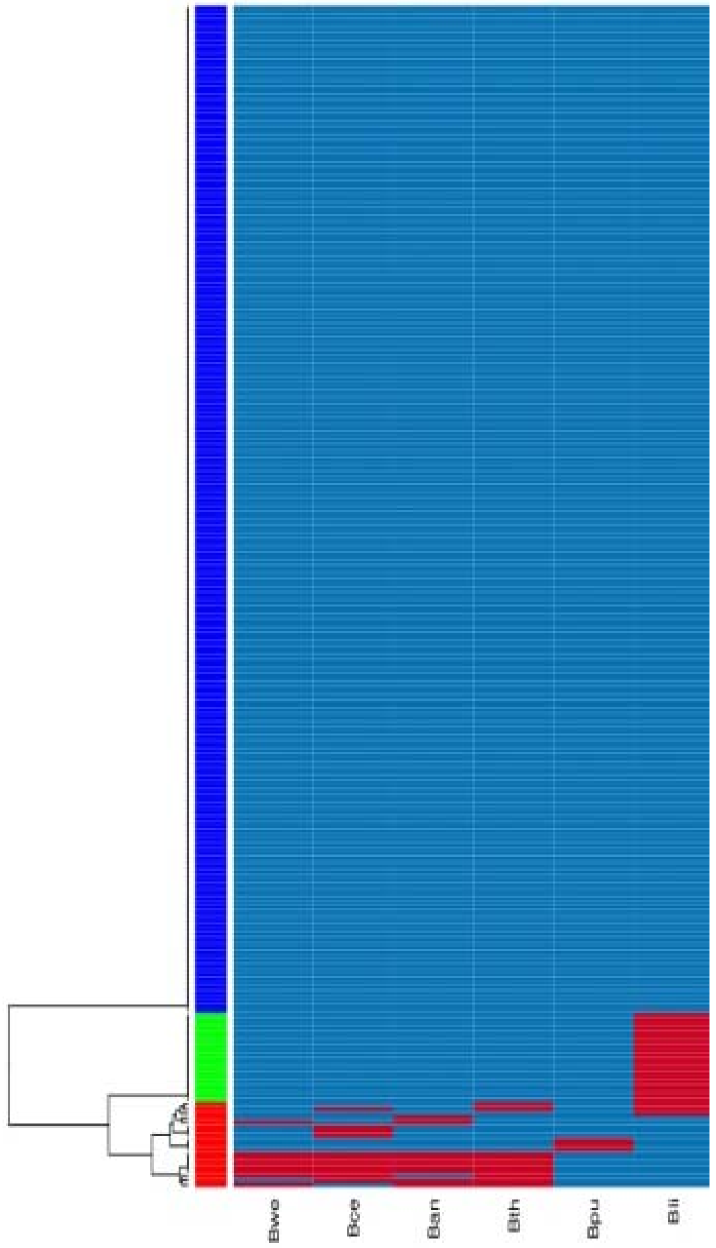

Figure S1. Phylogenetic profile of essential genes.
Blue indicates the presence of orthologous hits and red indicates the absence of detectable orthologous hits. Hierarchical clustering is applied by row. Essential genes are mostly conserved in the *B. subtlis* clade, and these hits are not informative for clustering analysis. Therefore, we exclude the *B. subtilis* hits from the hierarchical clustering analysis and heat map presentation.

(A) ω, coat genes

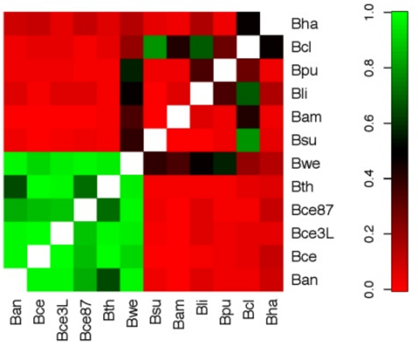

(B) dN, coat genes

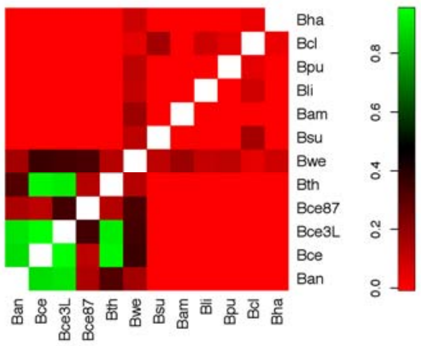

(C) dS, coat genes

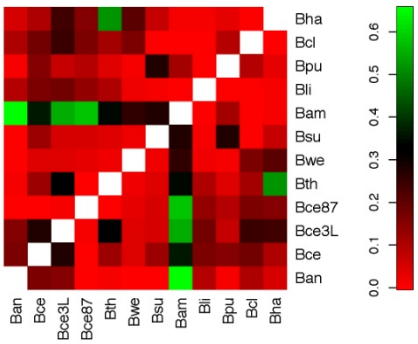

(D) ω, essential genes

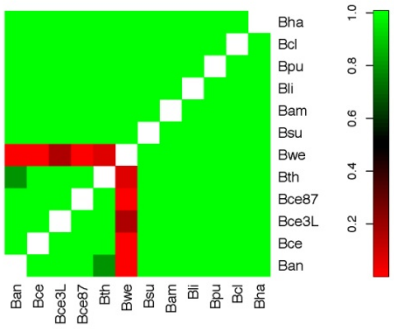

Figure S2. Heatmap presentations for all pairwise Wilcoxon tests for ω, dN, and dS with additional genomes.

Figure legends represent p-values. (A) ω, (B) dN, and (C) dS of coat protein genes to nonCE genes in *Bacillus* genomes. Alternative hypotheses: coat ω, dN, or dS > nonCE ω, dN, or dS. (D) Pairwise comparison of ω of essential genes to nonCE genes. Alternative hypothesis is essential gene ω > nonCE ω. In all figures, each cell in a heatmap is a p-value of a one-side Wilcoxon test. Data and codes used to perform the tests and generate the figures are at `https://github.com/hongqin/BacillusSporeCoat/tree/master/pairwise.matrix.omega.062310`.

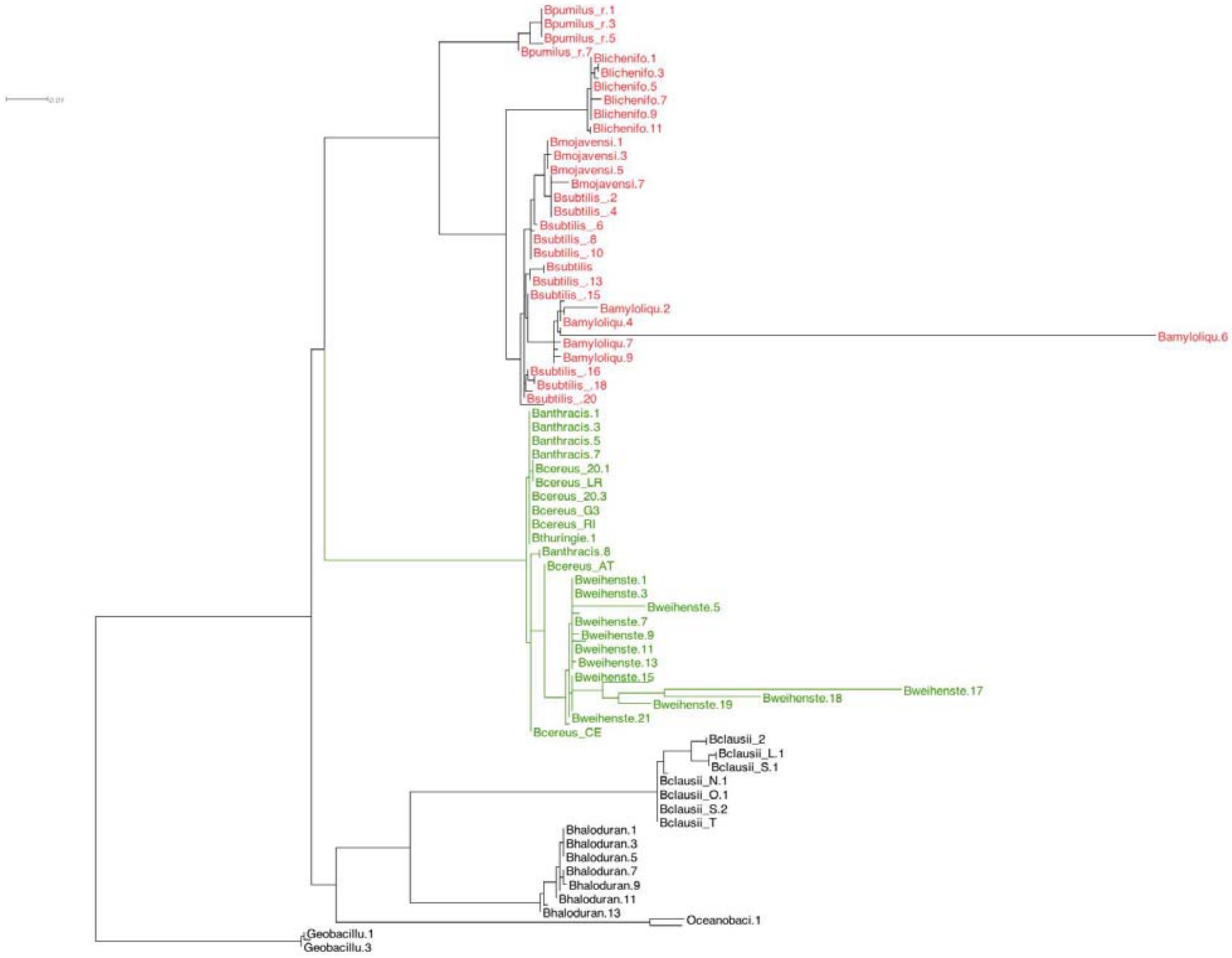

Figure S3. One of the maximal likelihood trees of 16S rRNA.

Red indicates the *B. subtilis*-clade, and green indicates the *B. cereus*-clade. Not all nodes are labeled due to space limit. Notice that 16S genes from many species are intermingled in the *B. cereus*-clade. Six trees were generated by maximal likelihood method in PAUP. None of them is a resolved species reference tree. The first tree output by PAUP is presented here. Three independent runs of the maximal likelihood inferences were performed.

The sequence alignment and PAUP command block is provided at `https://github.com/hongqin/BacillusSporeCoat/blob/master/bacillus.species.tree/16s.paup.053108.b.nexus`.

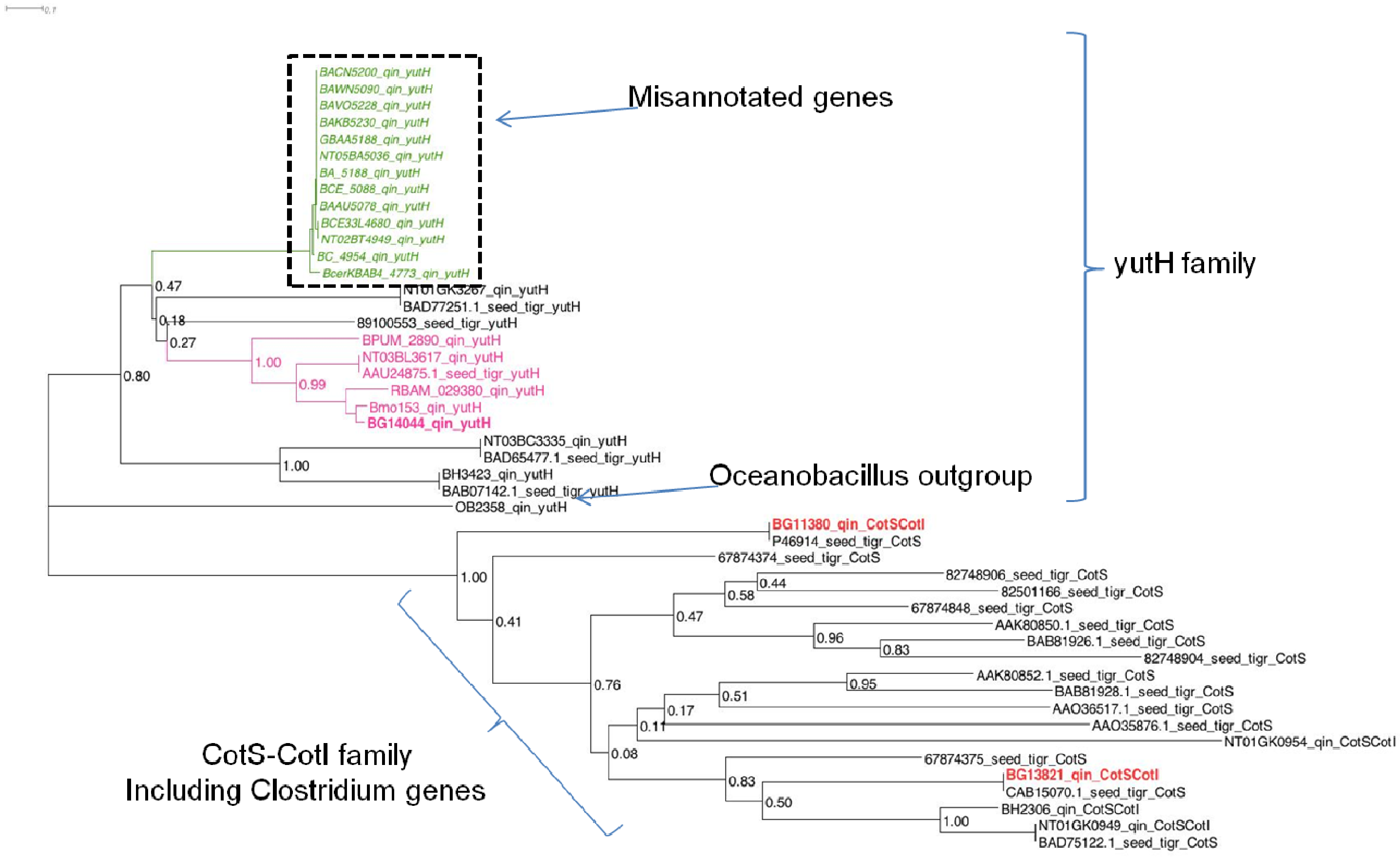

Figure S4. Bootstrap consensus tree of the CotS-CotI-YutH family.

The three *B. subtilis* gene members of this family are in bold font, BG14044, BG11380, and BG13821. The green-colored taxa in the doted box indicates the *yutH* genes that are misannotated as "*cotS*" in many *B. cereus*-group genomes (3 strains of *B. cereus*, one strain of *B. thuringiensis*, and 8 strains of *B. anthracis*). The pink-colored taxa are yutH members in the *B. subtilis*-group . Seed proteins for the TIGR CotS and YutH families for Hidden Markov Modeling are included for comparison (labeled as "seed tigr CotS" and "seed tigr YutH"). Proteins identified as CotS-CotI or YutH orthologs by us are labeled as "qin CotSCotI" and "qin YutH", respectively. The CotS family and the YutH family form two clades. Homologs of *Bacillus* genes from *Oceanobacillus* and *Geobacillus* species are included. The evolutionary history was inferred using the neighbor-joining method. The percentage of replicate trees in which the associated taxa clustered together in the bootstrap test are shown next to the branches. The tree is drawn to scale, with branch lengths in the same units as those of the evolutionary distances used to infer the phylogenetic tree. The evolutionary distances were computed using the JTT matrix-based method and are in the units of the number of amino acid substitutions per site. Phylogenetic analyses were conducted in MEGA4 and the tree is presented using Dendroscope.

Improved annotations of *Bacillus* spore coat proteins.

Our analyses of coat protein phylogeny and orthologous groups allowed us to place coat proteins into phylogenetically meaningful families, and to revisit and improve previous annotations, such as those in the genome databases and in previous studies [2]. About 50% of our orthologous profiles differ from those previously published, and in the case of six genes (cotI, cotS, cotSA, cotV, and ymaG) those profiles are inconsistent in various ways in at least four genomes. For example, we discovered orthologs of YmaG (BG13415) that were not reported previously. We note that low complexity regions (LCRs), which can be responsible for spurious homology hits, are present in YmaG (43% LCR, 53 out of 123 amino acids). We included LCRs in BLASTP searches and detected reciprocal-best-hits of YmaG with E-values less than $1x10^{-6}$ in all of the studied genomes. We argue that at least some of these hits are plausible YmaG orthologs. We believe that the inclusion of LCR sequence in the phylogenetic analyses (albeit with caveats) allows us to generate a more complete understanding of coat protein relatedness than previous studies.

We also generated improved coat protein annotations by more careful analysis of coat protein families. For example, CotI (BG13821, previously YtaA), CotS (BG11380) and YutH (BG14044) constitute a family, based on their similar sequences. Although our analysis indicates that CotI and CotS are not encoded in the *B. anthracis* and *B. cereus* genomes, both genes are annotated in those species. Noticeably, TIGR assigned some coat proteins, including the potential YutH orthologs of *B. thuringiensis*, to both YutH and CotS families using sequence similarity approaches. To better understand this discrepancy, we generated phylogenic trees using the CotS, CotI, and YutH sequences (Figure S4). The TIGR seed CotS and CotI proteins are from *Clostridia*, and they are grouped with BG11380 and BG13821, and we did find detectable orthologs of CotS and CotI in the other species of the *B. subtilis* clade. In contrast, the yutH clade resembles the species reference tree (see the pink and green labeled taxa in Figure S4). Because *B. subtlis* is a model organism, genes in other *Bacillus* species are often named after their counterparts in the *B. subtilis* genome. It appears that the annotated CotI and CotS orthologs in the *B. cereus* group genomes were named after a wrong *B. subtilis* gene member and should re-annotated as yutH.

Another interesting finding concerns YobN (BG13506). The two best matches in *B. licheniformis* to *B. subtilis* YobN are the contiguous genes NT02BL2036 and NT02BL2037, which are highly similar to the 5'and 3' portions of yobN, respectively. This unusual case did not pass the topology comparison and prompted us to examine it carefully. It turns out that a stop codon at the end of NT02BL2036 introduces an eight-residue gap in the sequence, "splitting" YobN into two ORFs. It is unclear at present whether this is due to a sequencing error or indicates a non-sense mutation with biological implications.

Analysis of sequence characteristics specific to a group of genes can reveal distinctive and shared functional features, as well as evolutionary commonalities. Compared to *B. subtilis* non-coat proteins, coat proteins are shorter than average in length (p=0.013, one-sided t-test). They contain an average of 6.8% LCRs, significantly more than do the non-coat proteins (with an average of 3.1%) at a p-value of 0.0088 (Wilcoxon rank sum test). Those coat proteins with a relatively large fraction of LCRs tend to be

relatively short, but this is the general trend of all of the proteins in *B. subtilis* (data not shown). Among the 73 coat proteins, 24 show disordered regions based on the consensus of three independent methods implemented in DisEMBL [3]. There is a moderate correlation between LCRs and disordered regions (R^2=0.11, p-value = 0.007), suggesting that only a small number of disordered regions in coat proteins are due to repeats.

The prevalence of disordered regions in coat proteins raises the possibility that these regions provide a function needed by most or all coat proteins. We do not know whether this is so, but we note that in some cases (such as the muscle protein titin [4]) disordered regions can act effectively as springs that unfold when stretched by an external force. If this were true for the disordered regions within coat proteins, then such regions could at least partially explain the ability of the coat to fold and unfold like the pleats of an accordion, since this dynamic action likely involves stretching and contracting the coat at specific points within each fold [5-11]. Interestingly, intrinsically disordered regions of proteins can evolve more rapidly than other regions, because disordered regions have fewer constraints than ordered regions [12]. However, because the alignment between sequences with intrinsic disorder may be poorer than other regions, evolutionary rates can appear faster than they actually are. Further analysis will be needed to determine whether these regions within coat proteins are, indeed, evolving faster than other regions.

List of the 34 essential *B. subtilis* genes for the species reference tree and topology tests

1	BG10101
2	BG10116
3	BG10130
4	BG10131
5	BG10221
6	BG10228
7	BG10229
8	BG10273
9	BG10279
10	BG10313
11	BG10327
12	BG10358
13	BG10422
14	BG10525
15	BG10532
16	BG10676
17	BG10957
18	BG10973
19	BG11004
20	BG11207
21	BG11384
22	BG11636
23	BG11638

24	BG11643
25	BG11711
26	BG11843
27	BG12084
28	BG12587
29	BG12687
30	BG12835
31	BG13128
32	BG13405
33	BG14048
34	BG19025

References

1. Moszer I, Jones LM, Moreira S, Fabry C, Danchin A: **SubtiList: the reference database for the *Bacillus subtilis* genome**. *Nucleic Acids Res* 2002, **30**(1):62-65.
2. Henriques AO, Moran CP, Jr.: **Structure, assembly, and function of the spore surface layers**. *Annu Rev Microbiol* 2007, **61**:555-588.
3. Linding R, Jensen LJ, Diella F, Bork P, Gibson TJ, Russell RB: **Protein disorder prediction: implications for structural proteomics**. *Structure* 2003, **11**(11):1453-1459.
4. Lee EH, Hsin J, von Castelmur E, Mayans O, Schulten K: **Tertiary and secondary structure elasticity of a six-Ig titin chain**. *Biophys J* 2010, **98**(6):1085-1095.
5. Chada VG, Sanstad EA, Wang R, Driks A: **Morphogenesis of bacillus spore surfaces**. *J Bacteriol* 2003, **185**(21):6255-6261.
6. Driks A: **The dynamic spore**. *Proc Natl Acad Sci U S A* 2003, **100**(6):3007-3009.
7. Plomp M, Leighton TJ, Wheeler KE, Pitesky ME, Malkin AJ: ***Bacillus atrophaeus* outer spore coat assembly and ultrastructure**. *Langmuir* 2005, **21**(23):10710-10716.
8. Plomp M, Leighton TJ, Wheeler KE, Malkin AJ: **The high-resolution architecture and structural dynamics of *Bacillus* spores**. *Biophys J* 2005, **88**(1):603-608.
9. Plomp M, Leighton TJ, Wheeler KE, Malkin AJ: **Architecture and high-resolution structure of *Bacillus thuringiensis* and *Bacillus cereus* spore coat surfaces**. *Langmuir* 2005, **21**(17):7892-7898.
10. Westphal AJ, Price PB, Leighton TJ, Wheeler KE: **Kinetics of size changes of individual *Bacillus thuringiensis* spores in response to changes in relative humidity**. *Proc Natl Acad Sci U S A* 2003, **100**(6):3461-3466.
11. Sahin O, Yong EH, Driks A, Mahadevan L: **Physical basis for the adaptive flexibility of Bacillus spore coats**. *J R Soc Interface* 2012, **9**(76):3156-3160.
12. Brown CJ, Johnson AK, Daughdrill GW: **Comparing models of evolution for ordered and disordered proteins**. *Mol Biol Evol* 2010, **27**(3):609-621.
